# Comparison of the sex hormones’ serum level in women with recurrent aphthous stomatitis and healthy population: a cross-sectional study

**DOI:** 10.1186/s12903-021-01812-9

**Published:** 2021-10-27

**Authors:** Fatemeh Lavaee, Zahra Ranjbar, Mina Jalalian, Mohammad Amin Amiri

**Affiliations:** 1 Oral and Dental Disease Research Center, Oral and Maxillofacial Disease Department, Shiraz University of Medical Sciences, Ghasr-dasht Street, Shiraz, Iran; 2 Student Research Committee, Shiraz University of Medical Sciences, Shiraz, Iran

**Keywords:** Recurrent aphthous stomatitis, FSH, LH, Prolactin, Testosterone, Estradiol

## Abstract

**Background:**

In this study, we aimed to evaluate the sex hormonal serum level in patients with recurrent aphthous stomatitis and compare them with healthy participants.

**Methods:**

This cross-sectional study was done on patients with recurrent aphthous stomatitis who had referred to Shiraz Dental Faculty, Oral and Maxillofacial Medicine Department during 2018–2019. The non -menopause women with recurrence of at least 3 lesions per year were enrolled in this study. The mean serum level of FSH, LH, PRL (prolactin), testosterone, DHT (Dihydrotestosterone), DHEA-S (Dehydroepiandrosterone sulfate), estradiol and progesterone of 30 participants in each group of case and control were measured and compared. The data were analyzed by SPSS version 18 and independent T-test, Mann–Whitney U test, Spearman’s correlation coefficient test, Chi-square test and Fisher’s test.

**Results:**

The mean serum level of DHEA-S in patients with recurrent aphthous stomatitis (RAS) was significantly lower than the control group (*p* value = 0.02). In addition to DHEA-S, the mean serum level of testosterone was lower in the evaluation group although this difference was not significant (*p* value = 0.057). Considering the effect of age on the mean serum level of sex hormones, our results revealed that only DHEA-S mean serum level was decreased by increasing the age of participants in patients with RAS (*p* value = 0.018). The number of participants with abnormal range of testosterone (*p* value < 0.0001) and progesterone (*p* value = 0.037) serum level was significantly more in patients with RAS. The frequency of RAS in a year did not show a significant relationship with the serum level of the evaluated hormones.

**Conclusion:**

The patients with RAS had a lower serum level of DHEA-S. The mean serum level of testosterone and progesterone was significantly abnormal in RAS patients.

**Supplementary Information:**

The online version contains supplementary material available at 10.1186/s12903-021-01812-9.

## Background

The oral mucosa can be affected by sex hormones fluctuation during puberty, pregnancy and different phases of menstruation cycle and menopause [1]. For instance, increasing the prevalence of recurrent aphthous stomatitis (RAS) a few days before menstruation has been reported in previous evaluations [2].

Recurrent aphthous stomatitis (RAS) is the most recurrent oral lesion with a prevalence range of 5–25% in different population. The pathogenesis of RAS is not clearly known. It is a painful yellowish ulcer with an erythematous border which is self-limited [3, 4].

Alteration in cellular immune response is detected for RAS pathogenesis. Function of sex hormones during menstrual cycle can be attributed to this immunologic changes [5]. On the other hand, the effect of sex hormones on other autoimmune diseases is known. The women’s strong tendency for autoimmune disease susceptibility may be attributed to the gender differences in hormonal secretion [5].

Sex steroid may affect DNA of the lymphocytes and modify their function, alter surface antigen expression, and interfere with cellular function [6]. The effect of sex hormones on immune function has been described in literature.

An interrelation between DHEA-S (Dehydroepiandrosterone sulfate) and immune mediators regulate leukocyte function. DHEA-S can regulate cytokine release and Th1 and Th2 secretion [7]. Progesterone is a natural immune suppressor which can affect immunologic mediators such as prostaglandin [8]. Testosterone is immunosuppressive and has anti-inflammatory effects [9].

In this study we aimed to evaluate the sex hormones profile with RAS in comparison to healthy population.

## Methods

This cross-sectional study was done on patients with RAS who referred to Shiraz Dental Faculty, Oral and Maxillofacial Disease Department during 2018–2019. The protocol of this study which was conducted according to the ethical principles of Helsinki [10], was approved by the ethics committee of Shiraz University of Medical Sciences (IR.SUMS.REC.1398.533).

The non-menopausal women with history of recurrent aphtous lesions (at least 3 recurrences in a year) which is confirmed by the researcher as an oral and maxillofacial medicine specialist have been enrolled in this study. The patients who were pregnant or had any other autoimmune diseases, those who had received systemic corticosteroid during previous 1 months or any other medication which can affect the sex hormone including contraceptives, and the participants with any other disease or problem which can affect the serum level of hormones were excluded from the study.

The healthy patients who referred to Shiraz Dental Faculty, Oral and Maxillofacial Disease Department for dental examination and had no history of RAS were enrolled in the control group. The participants in the case and control groups were matched by age and menstruation.

In each group of case and control, 30 participants with normal menstrual cycle of 28–30 days were recruited. After they had signed the written consent form, 5 cc blood sample from both groups was taken on day 3 of menstruation period for non-menopausal participants. The mean serum level of hormones follicle stimulating hormone [FSH] (gamma kit from Iranian Padtan Gostar Isar company), Luteinizing hormone [LH] (gamma kit from Iranian Padtan Gostar Isarcompany), prolactin (gamma kit from Iranian Padtan Gostar Isarcompany), testosterone (elisa kit from the United States monoband company), dehydrotestosterone [prl] (elisa kit from the United States monoband company), dehydroepiandrosterone-sulfate [DHEA-S] (elisa kit from the United States monoband company), estradiol (elisa kit from the United States monoband company) and progesterone (elisa kit from th e United States monoband company) were measured and compared between the two groups in Motahari laboratory. The relationship of the serum level of these hormones and age, recurrence rate was assessed. The demographic data including age and recurrence of RAS in all patients were registered. The data were analyzed by SPSS version 18 and independent T-test was used for comparing the mean age of the case and control groups. Mann–whitney U test was used for comparing the serum level of sex hormones in the case and control groups. Spearman’s correlation coefficient was used to find the relationship of age and the frequency of RAS with sexual hormones. Logistic regression (backwards: LR) was used for controlling the effect of age.

## Results

30 participants were enrolled in the evaluation and control groups. The mean age of the participants with RAS and healthy controls were 33.53 ± 7.59 and 34.00 ± 7.41 years, respectively. The participants’ age in both groups were matched (*p* value = 0.811).

The mean serum levels of sex hormones in the evaluated group are presented in Table 1.

**Table 1:** The serum level of sex hormones in patients and control group

Hormones	Patient				Control				P value
	N	Mean	Standard deviation	Median	N	Mean	Standard deviation	Median	
FSH (mIU/ml)	30	8.01	5.17	7.32	30	8.03	9.99	5.34	n.s
LH (mIU/ml)	30	4.78	3.43	3.54	30	5.87	7.34	3.64	n.s
PRL (ng/ml)	30	17.02	10.29	14.8	30	14.46	5.62	13.91	n.s
TESTO (ng/ml)	30	0.54	1.04	0.2	30	0.38	0.24	0.3	0.057*
DHEA-S (μg/ml)	29	1.06	0.67	0.8	30	1.49	0.6	1.4	0.02
EST (pg/ml)	30	77.02	75.63	63.1	28	80.05	40.68	79.8	n.s
PROG (ng/ml)	30	0.77	0.73	0.55	30	0.47	0.30	0.43	n.s
DHT (pg/ml)	30	332.41	132.03	338.65	29	339.36	323.64	247.00	n.s

DHT dihydrotestostrone, DHEA-S Dehydroepiandrosterone sulfate, EST estrogen, PRL prolactone, PROG progesterone, TESTO testostrone, n.s. nonsignificant

^*^Statistically not significant but considerable

The comparison of these values between patients with RAS and participants in the control group are also reported in Table 1 (Additional file 1). According to these results, DHEA-S serum level was significantly more in the control participants than patients with RAS (*p* value = 0.020). In patients with RAS, DHEA-S was decreased by aging amongst the participants (*p* value = 0.018). In the control participants, progesterone increased by increase in the age of participants (*p* value = 0.007) (Additional file 2).

The percentage of participants with abnormal range of sex hormones is described in Table 2.

**Table 2:** The percentage of the participants with abnormal range of sex hormones and correlation with RAS

Hormones	Case		Control		*p* value
	Count	% within group	Count	% within group	
FSH 2.5–13.2 (mIU/ml)	6	20.0	6	20.0	n.s
LH (1–7.9 mIU/ml)	5	16.7	4	13.3	n.s
PRL (3.5–17.9 ng/ml)	9	30.0	8	26.7	n.s
TESTO (0.2–0.95 ng/ml)	17	58.6	4	13.3	< 0.0001
DHEA-S (0.37–2.71 μg/ml)	4	13.8	1	3.3	n.s
EST (9-175 pg/ml)	2	6.7	1	3.6	n.s
PROG (0.15–1.4 ng/ml)	11	36.7	4	13.3	0.037
DHT (24-368 pg/ml)	10	33.3	5	17.2	n.s

PRL prolactone, TESTO testosterone, DHT dihydrotestostrone, DHEA-S dehydroepiandrosterone sulfate, EST estrogen, PROG progesterone, n.s. nonsignificant

Except testosterone and progesterone in patients with RAS, the prevalence of participants with the normal range of hormones between the evaluated and control groups was not significantly different.

The frequency of RAS in a year had no significant relationship with the serum level of the evaluated hormones (Table 3).

**Table 3:** The frequency of RAS and relationship with sex hormones

Hormones	Variables	Number
FSH (mIU/ml)	*p* value	n.s
	Correlation coefficient	0.27
LH (mIU/ml)	*p* value	n.s
	Correlation coefficient	0.07
PRL (ng/ml)	*p* value	n.s
	Correlation coefficient	0.29
TESTO (ng/ml)	*p* value	n.s
	Correlation coefficient	0.22
DHEA-s (μg/ml)	*p* value	n.s
	Correlation coefficient	-0.13
EST (pg/ml)	*p* value	n.s
	Correlation coefficient	-0.15
PROG (ng/ml)	*p* value	n.s
	Correlation coefficient	0.04
DHT (pg/ml)	*p* value	n.s
	Correlation coefficient	-0.21

DHT dihydrotestostrone, DHEA-S Dehydroepiandrosterone sulfate, EST estrogen, PRL prolactin, PROG progesterone, TESTO testosterone, n.s. nonsignificant

Logistic regression has been used in order to analyses the effect of hormones and controlling the confounding factors such as age. The frequency of RAS has been divided into more than 3 times frequency in a year and less than 3 times in a year. The effect of hormones on the frequency of RAS has been assessed by logistic regression analysis. According to the univariate logistic regression analysis, by one unit increase in DHT, the risk of having more frequent RAS (> 3 time RAS in a year) decreases by 4% (*p* value: 0.04, odds ratio 0.96, 95% confidence interval 0.94–0.99).

Moreover, the effect of hormones on the risk of RAS for all participants, patients with history of RAS and control group, have been assessed by logistic regression. According to the univariate logistic regression, by one-unit increase in DHEA-S, the risk of RAS occurrence, 0.68 time for adjusted OR decreases (*p* value: 0.03; odds ratio 0.32, 95% CI 0.12–0.9).

## Discussion

According to the results of the present study amongst all the evaluated hormones, only the mean serum level of DHEA-S in patients with RAS was significantly lower than the control group. In addition to DHEA-S, the mean serum level of testosterone was lower in the evaluation group although this difference was not significant.

Considering the effect of age on the mean serum level of sex hormones, our results revealed that only DHEA-S mean serum level was decreased by increase in the age of participants in patients with RAS.

The number of participants with abnormal range of testosterone and progesterone serum level was significantly higher in patients with RAS. Serum level reduction in DHT and DHEA-S increase the risk of RAS occurrence and its frequency.

Previous studies have shown some relationships between steroid concentration reduction and some autoimmune diseases including rheumatoid arthritis and systemic lupus erythematosus [11]. Some similar studies reported androgen deficiency (DHT, DHEA-S, etc.) in patients with sjogren syndrom [12, 13], rheumatoid arthritis [14], and systemic lupus erythematosus [15, 16]. In addition, Forsblad-d’ Elia et al. prescribed 50 mg oral DHEA daily for women with sjogren syndrome; they reported dry mouth symptom improvement [12]. Taylor et al. concluded that acute stress can elevate the serum levels of DHEA-S as cortisol does [17]. Anete Rejane et al. in an evaluation reported no significant difference in the salivary level of DHEA-S between patients with RAS and healthy controls [18]. This result was confirmed for oral lichen planus patients in another study [19].

In the present study, the DHEA-S serum level was significantly lower in patients with RAS in comparison to the controls. Also in the RAS group, DHEA-S serum level was decreased by aging. This is noticeable since all the participants were premenopausal and other evaluated hormones did not show significant age-related changes. DHEA-S is a precursor for androgens and estrogen. This steroid which is abundant in the human body is secreted by the adrenal cortex, CNS and gonads. The prohormones DHEA, DHEA-S which can be produced in the peripheral tissue play an important role in women’s health [20]. IL-2 (Th1 secretion) increment and IL-6 and IL-10 reduction are the most prominent effects of this steroid. A fall in DHEA-S concentration can play an important role in the pathogenesis of some immunologic diseases [7].

Previous studies have assessed the menstrual cycle and its hormonal changes in patients with RAS. Some studies reported more frequent RAS lesions in the luteal phase in which progesterone increment plays an important role [21]. In accordance to what they assumed, the results of this study showed more abnormality in the progesterone and testosterone level of patients with RAS in comparison with healthy controls.

Some other studies did not reveal any relationship between the premenstrual phase and RAS. However, low level of progesterone has been reported in patients with mild and severe RAS [22–24]. Corpus luteam secretes progesterone during the second half of the menstrual cycle. This hormone is at its peak in the third week of menstrual cycle [25]. Any abnormality in this hormone’s serum level can affect the immunologic function. Some studies reported more prevalent occurrence of RAS in the luteal phase [21]; they did not measure the serum level of this hormone. They just assessed the time association of RAS and menstrual cycle. However, in other studies decrease in the serum level of progesterone has been associated with more RAS [22–24]. Evaluating the serum level of progesterone in different phases of the menstrual cycle can reveal the exact role of progesterone changes and incidence of RAS.

There are some controversies about the role of testosterone in patients with auto-immune diseases. According to the literature testosterone has controversial effects on the systemic lupus erythematosus (SLE) complications in a different phase of disease [26]. Also, another evaluation did not show noticeable SLE symptoms improvement by testosterone prescription [27]. In the literature, there are some reports about the protective role of testosterone in SLE and Rheumatoid arthritis and Multiple sclerosis patients [28, 29]. In the present study, the mean serum level of testosterone in the healthy control group was more than patients with RAS; however, it was not significant. Increasing the sample size may reveal this difference more precisely. Testosterone is primarily secreted by the gonads and adrenal cortex in both sexes. Also, testosterone is synthesized from cholesterol in the brain (neurosteroid) [30]. The proportion of its secretion changes by aging, in the fourth decade of a woman’s life, it is in half of its secretion amount [31]. Testosterone can inhibit the effect of cyclooxygenase pathway of arachidonic acid metabolism by prostaglandin secretion inhibition. Testosterone reduction can decrease the regulatory T cells expansion, which is important in controlling auto reactive T cells and B cells expression [32].

Since the menopausal condition has a major effect on the level of sex hormones in this study we considered the similar non-menopause condition of all participants of both evaluated groups. However, environment and some other factors such as BMI can affect hormonal change and this should be considered.

The mean serum levels of FSH, LH, Prolactin, Testosterone, Estradiol and DHT have not shown any significant change by increasing the age of participants. However, DHEA-S has decreased in non-menopause participants of patients with RAS. Considering the decreasing effect of age on this hormone, in the control group, progesterone increased by aging which is challenging.

Evaluating a larger sample size of patients with RAS and assessing their sex hormones in different parts of the menstrual phase and even in postmenopausal women is recommended for future researches. Also it can be suggested to consider and assess all effective factors including BMI and environmental situations.Since sex hormone can be affected by BMI. Sexual steroid hormones can play acausetive role for metabolic disorders and obesity.

Assessing these hormonal changes in males is also suggested. Revealing the effect of these hormones can introduce new methods of RAS prevention or treatment.

## Conclusion

The patients with RAS had lower serum level of DHEA-S in spite of non-menopausal situation of all participants. DHEA-S mean serum level was decreased by aging in contrast to the healthy controls in which the mean value was increased. The mean serum levels of testosterone and progesterone were significantly abnormal in RAS patients. The frequency of RAS in a year has no relationship with the serum level of the evaluated hormones. In addition, serum level reduction in DHT and DHEA-S, increase the risk of RAS occurrence and its frequency.

## Supplementary Information

**Additional file 1:** For future reference.

**Additional file 2:** Relationship of the evaluated hormones and age.

## Data Availability

The datasets used and/or analyzed during the study are available from the corresponding author on reasonable request.

## Abbreviations

CNS: Central nervous system
DHEA-S: Dehydroepiandrosterone sulfate
DHT: Dihydrotestosterone
EST: Estrogen
FSH: Follicle stimulating hormone
LH: Luteinizing hormone
PRL: Prolactin
PROG: Progesterone
RAS: Recurrent aphthous stomatits
