# Supplementary material for: Comparison of the sex hormones’ serum level in women with recurrent aphthous stomatitis and healthy population: a cross-sectional study
Source: BMC Oral Health. 2021 Oct 27;21:551. doi: 10.1186/s12903-021-01812-9 (PMC8549300; doi:10.1186/s12903-021-01812-9)
Supplement: Supplementary file 2 — Additional file 2: Relationship of the evaluated hormones and age. [file 12903_2021_1812_MOESM2_ESM.docx]

| Groups | Hormones | Correlation Coefficient | P value |
| --- | --- | --- | --- |
| Patients | FSH (mIU/ml) | 0.127 | n.s. |
|  | LH (mIU/ml) | 0.140 | n.s. |
|  | PRL (ng/ml) | 0.114 | n.s. |
|  | TESTO (ng/ml) | -0.151 | n.s. |
|  | DHEA-S (μg/ml) | -0.438 | 0.018 |
|  | EST (pg/ml) | 0.107 | n.s. |
|  | PROG (ng/ml) | 0.281 | n.s. |
|  | DHT (pg/ml) | -0.087 | n.s. |
| control | FSH (mIU/ml) | 0.223 | n.s. |
|  | LH (mIU/ml) | 0.010 | n.s. |
|  | PRL (ng/ml) | -0.249 | n.s. |
|  | TESTO (ng/ml) | -0.020 | n.s. |
|  | DHEA-S (μg/ml) | -0.044 | n.s. |
|  | EST (pg/ml) | 0.091 | n.s. |
|  | PROG (ng/ml) | 0.485 | 0.007 |
|  | DHT (pg/ml) | -0.352 | n.s. |

Relationship of the evaluated hormones and age

The relationship of the evaluated hormones and age of participants in each group is demonstrated in this supplementary Table.

DHT: Dihydrotestosterone, DHEA-S: Dehydroepiandrosterone Sulfate,

EST: Estrogen, PRL: Prolactin, PROG: Progesterone, TESTO: Testosterone, n.s.: Not Significant
